# *Faecalibacterium duncaniae*-derived metabolites protect intestinal epithelial integrity under inflammatory conditions in dairy calves

**DOI:** 10.1128/aem.00854-26

**Published:** 2026-07-24

**Authors:** Dongxiao Du, Yunlong Gao, Pengchao Zhan, Yan Wang, Shengyong Mao, Smerjai Bureenok, Sophany Morm, Ahmed E. Kholif, Tarek Morsy, Zan Huang, Jinxin Liu

**Affiliations:** 1 Laboratory of Gastrointestinal Microbiology, College of Animal Science and Technology, Nanjing Agricultural University 524556, Nanjing, China; 2 Jiangsu Key Laboratory of Gastrointestinal Nutrition and Animal Health, National Center for International Research on Animal Gut Nutrition, College of Animal Science and Technology, Nanjing Agricultural University 524556, Nanjing, China; 3 Faculty of Agricultural Innovation and Technology, Rajamangala University of Technology Isan 202946, Nakhon Ratchasima, Thailand; 4 Department of Animal Science, Faculty of Agriculture and Food Processing, National University of Battambang 422302, Battambang, Cambodia; 5 Department of Animal Sciences College of Agriculture and Environmental Sciences, North Carolina A&T State University 3616, Greensboro, North Carolina, USA; 6 Dairy Science Department, National Research Centre 68787, Giza, Egypt; Universita degli Studi di Napoli Federico II, Portici, Italy

**Keywords:** colon organoid, inflammatory bowel disease, *Faecalibacterium duncaniae*, anti-inflammatory

## Abstract

Preweaning diarrhea and intestinal inflammation represent leading causes of morbidity and economic loss in modern dairy calf production systems, causing impaired growth performance, increased mortality, and substantial veterinary costs that collectively constrain farm profitability. *Faecalibacterium duncaniae*, a key butyrate-producing commensal bacterium, is consistently depleted in diarrheic dairy calves, yet its protective effects on the ruminant intestinal epithelium remain mechanistically understood. In this study, a long-term expandable ruminant colonic organoid model was used to investigate the effects of *F. duncaniae*-derived metabolites under inflammatory conditions induced by tumor necrosis factor-α (TNF-α). TNF-α stimulation caused epithelial injury characterized by elevated inflammatory cytokine expression, impaired barrier function, and increased epithelial permeability. Treatment with *F. duncaniae* cell-free supernatant markedly attenuated inflammatory responses, restored epithelial proliferation, and preserved tight junction integrity, as evidenced by reduced permeability and increased expression of barrier-related proteins. Transcriptomic analysis further indicated suppression of inflammation-associated signaling pathways, including chemokine signaling and NF-κB pathways, accompanied by enrichment of energy metabolism and epithelial repair pathways, thereby creating a molecular environment conducive to epithelial barrier improvement and repair. In addition, comparison with the bacterial culture medium and the single metabolite sodium butyrate (NaB) further supported the role of *F. duncaniae*-derived metabolites in barrier repair. Collectively, these findings demonstrate that *F. duncaniae*-derived metabolites protect intestinal epithelial integrity under inflammatory stress and provide mechanistic and translational support for microbiota-targeted nutritional strategies to improve gut health in dairy calves, thereby establishing a foundation for the development of postbiotic-based interventions aimed at reducing diarrhea incidence, decreasing antimicrobial dependence, and enhancing growth efficiency and long-term productivity in dairy systems.

**IMPORTANCE:**

Dairy calves are highly susceptible to severe impacts from diarrhea and intestinal inflammation before weaning, leading to malnutrition, dehydration, and even death. The costs associated with enteritis, including mortality, productivity losses, and treatment expenses, have been rising annually. Therefore, the treatment and prevention of enteritis are of paramount importance. *Faecalibacterium duncaniae*, a beneficial bacterium that produces butyrate, plays a crucial role in maintaining gut health. However, its specific protective mechanisms in intestinal inflammation remain poorly understood. This study reveals the molecular mechanisms by which *F. duncaniae* metabolites protect the intestinal barrier under inflammatory stress, providing valuable insights into how microbiota-based nutritional interventions can enhance gut health in neonatal and preweaning dairy calves. These strategies may offer innovative approaches to managing calf intestinal diseases, improving their health and growth, and enhancing overall productivity in dairy farming.

## INTRODUCTION

Gastrointestinal disorders, particularly preweaning diarrhea and intestinal inflammation, remain major health challenges in dairy calf production (1, 2), leading to impaired growth performance, increased morbidity, and substantial economic losses (3, 4). During early life, the gastrointestinal tract of dairy calves is functionally immature, characterized by underdeveloped epithelial barrier function and unstable host–microbiota interactions, which predispose calves to inflammation, infection, and nutrient malabsorption (5, 6). Among intestinal segments, the colon plays a critical role in microbial fermentation and immune regulation during early development (7). However, its structural and functional immaturity renders it particularly vulnerable to inflammatory insults. Consequently, colitis-like intestinal inflammation has emerged as a key constraint on calf health and productivity (8, 9), while current preventive and therapeutic strategies remain limited in efficacy (10). Although conventional animal models and two-dimensional cell cultures have provided valuable insights into intestinal inflammation, the inaccessibility of the gastrointestinal tract *in vivo* greatly limits detailed investigation of host–microbiota interactions in ruminants, and traditional cells cannot recapitulate the three-dimensional structure and functional complexity of ruminant intestinal epithelium (11, 12).

Recent advances in organoid technology have provided physiologically relevant *in vitro* platforms for investigating intestinal epithelial function and host–microbe interactions (13). Intestinal organoids derived from stem cells self-organize into three-dimensional structures that recapitulate key features of native intestinal tissue, including epithelial differentiation, barrier formation, and cytokine responsiveness (14). These models have been widely applied in human and mouse systems; however, in ruminants, *in vitro* models capable of reflecting colonic characteristics are still lacking. Therefore, the development of ruminant colonic organoids not only preserves the three-dimensional structure and functional properties of the colonic epithelium but also provides a controllable and reproducible experimental platform for studying inflammatory stress, barrier function, and host–microbiota interactions, offering significant value for investigating the physiological and pathological mechanisms of the ruminant colon (15). In ruminant research, organoid systems have been successfully established from gastric and small intestinal tissues (16, 17); however, colonic organoid models, particularly those suitable for long-term expansion and mechanistic studies, remain poorly developed. Establishing a robust ruminant colonic organoid model is therefore essential for advancing mechanistic understanding of intestinal inflammation relevant to dairy production. Nutritional and microbiota-based interventions have gained increasing attention as sustainable strategies to improve intestinal health and disease resistance in dairy calves (18, 19). Probiotics and their metabolites are known to modulate immune responses, enhance epithelial barrier integrity, and promote intestinal homeostasis (15, 20). *Faecalibacterium duncaniae* (formerly *Faecalibacterium prausnitzii*) (21), a dominant butyrate-producing commensal bacterium (22), has been consistently associated with intestinal health across mammalian species (23). Importantly, reduced abundance of *F. duncaniae* has been observed in calves experiencing diarrhea, suggesting a conserved link between its depletion and intestinal inflammation (24). Its metabolites, particularly butyrate, have been shown to exert anti-inflammatory effects and maintain intestinal barrier function (25). However, the epithelial mechanisms underlying these effects in ruminants remain incompletely understood.

In the present study, we established a long-term expandable colonic organoid model from ruminant intestinal tissue and constructed a reproducible inflammatory injury model using tumor necrosis factor-α (TNF-α). Using this platform, we investigated the protective effects of *F. duncaniae*-derived metabolites on intestinal epithelial inflammation and barrier dysfunction. By integrating epithelial phenotyping with transcriptomic analysis, we aimed to elucidate the cellular pathways involved in epithelial injury and repair under inflammatory stress. These findings provide mechanistic insight into microbiota-derived regulation of intestinal epithelial integrity and support the development of microbiota-based nutritional strategies to improve gut health in dairy calves.

## MATERIALS AND METHODS

### Animals and sample collection

#### Cohort 1

The colon tissue used in this study was obtained from healthy male Hu sheep aged 2–3 months. Ovine intestinal tissue was selected as the organoid source based on the anatomical and functional similarities between sheep and cattle colonic epithelium, the relative ease of tissue collection, and its established utility in ruminant organoid research.

#### Cohort 2

At Dingzhuang Farm in Jurong, Jiangsu Province, a cohort of six newborn Holstein male calves was housed in a calf barn. Within 10 h of birth, the calves were fed colostrum twice (first 4 L, then 2 L). Subsequently, the calves were fed twice daily according to their body weight and were gradually introduced to solid feed starting on day 7. The calves had free access to water, and the temperature of the milk and water provided was maintained between 35°C and 37°C. No therapeutic or prophylactic antibiotics were administered to the calves. Fecal samples were collected from the calves by rectal sampling on days 1, 3, 7, 13, and 28 before the early feeding (*N* = 30). Additionally, to investigate the correlation between the gut microbiota and disease, we also collected fecal samples from 20 healthy calves and 20 diarrheic calves and then stored them in cryovials at −20°C, followed by transfer to a −80°C freezer for long-term storage (*N* = 40).

After thawing, DNA extraction was carried out following the protocol from Wang et al. (26) using repeated bead beating with a mini-bead beater (Biospec Products, Bartlesville, OK, USA).

### Shotgun metagenomics and taxonomic profiling

Thirty longitudinal rectal samples from six calves in the first month, along with 40 rectal samples from diarrheic and healthy calves, were used for metagenomic sequencing. The metagenomic shotgun sequencing libraries were constructed and sequenced by Shanghai Biozeron Biological Technology Co., Ltd. Briefly, for each sample, metagenomic libraries were prepared according to standard genomic DNA library preparation protocols. Library concentrations were measured using the High Sensitivity Double-Stranded DNA Kit on a Qubit Fluorometer (Thermo Fisher Scientific, USA). Sequencing was performed on the Illumina platform (PE150 mode) following standard procedures. The data of rectal samples from 30 early-life calves are shown in Data S1. The raw sequence reads were processed with quality trimming using Trimmomatic (version 0.35) (27) to remove adapter contamination and low-quality reads. Bowtie2 (version 2.4.2) (28) was used to remove reads aligning to the bovine genome (version ARS-UCD1.3). The reads that underwent host-genome contamination removal and quality filtering were retained as clean reads for further analysis.

Species-level profiling and relative abundance were determined using Sylph (29) with the GTDB r220 reference database. Longitudinal associations of gut microbial species abundance over time and differences in microbial species abundance between healthy and diarrheic calves were assessed using Microbiome Multivariable Associations with Linear Models (MaAsLin2) in R (30). The relative abundance of *F. duncaniae* was analyzed in relation to the age of dairy calves using a linear regression model. The data used for analysis were obtained from the *F. duncaniae* data set, excluding an outlier sample. Statistical analysis was conducted using the lm() function in R. Differences in β-diversity were evaluated using adonis2 (permutational multivariate analysis of variance test) with the vegan package in R.

### Crypt isolation

Following the method established by Sato et al. (31), approximately 5–7 cm segments of colon tissue, spanning from the transverse to the descending colon, were collected. The tissues were excised with sterile surgical tools and immersed in cold Dulbecco’s phosphate-buffered saline (DPBS) containing 100 U/mL penicillin/streptomycin (bioGenous, Hangzhou, China). The serosal surface was exposed by open abdominal incision, and the colon was longitudinally cut. The lumen was rinsed with DPBS to remove debris and microbes, then placed in sterile culture dishes. Most mucus was gently removed with a slide; tissues were then resuspended in DPBS with antibiotics. Repeated centrifugation, aspiration, and resuspension were performed to remove residual mucus. Crypts were released by incubating the tissue in 2 mM ethylenediaminetetraacetic acid (EDTA; Beyotime Biotechnology, Shanghai, China) at 4°C on a shaker at 80 rpm for 30 min. The crypts were gently shaken loose, filtered through a 100 μm mesh, and assessed under microscopy. The supernatant containing crypts was centrifuged at 1,000 rpm for 2 min and resuspended in advanced DMEM/F12 medium (Gibco, USA) containing 0.1% bovine serum albumin (BSA; Sigma-Aldrich, USA), 2.7% gentamicin, and 1% penicillin/streptomycin.

### Intestinal organoid culture

The protocol for 3D organoid culture has been described previously (32). A total of 200–1000 crypts were resuspended in 100 μL of advanced DMEM/F12 and mixed with 200 μL of Matrigel (BD Biosciences, USA). Droplets (~50 µL) of this mixture were seeded into 24-well plates and incubated at 37°C for 30 min to allow gelation. Subsequently, 500 μL of prewarmed complete IntestiCult growth medium (STEMCELL Technologies, Canada) was added. The plates were cultured at 37°C with 5% CO_2_, with media changes every 2–3 days. Organoids generally formed within 8–9 days and were imaged regularly.

### Organoid passaging

When the organoids reached days 8–9 of culture, the IntestiCult medium was removed from the cultured organoids, and the Matrigel matrix was dissolved by replacing it with 1 mL ice-cold DPBS. The resuspended organoids were transferred to a 15 mL centrifuge tube, and the DPBS volume was increased to 6 mL. The sample was centrifuged at 1,000 rpm for 2 min at 4°C to precipitate the organoids, and the supernatant was then removed. The organoids were resuspended in 1 mL TrypLE Express (Thermo Fisher Scientific) and incubated at 37°C for 1 min. The number of cell clusters was counted under the microscope, and the sample was diluted to a concentration of 200–1,000 crypts per 100 µL. The diluted sample was subsequently mixed with Matrigel and inoculated into new tissue culture plates for further experiments.

### Organoid cryopreservation

When organoids reached a diameter of >100 µm, the IntestiCult medium in the cultured organoid system was removed, and the Matrigel matrix was dissolved by replacing it with 1 mL of ice-cold DMEM/F12 medium (bioGenous). The resuspended organoids were transferred to a microcentrifuge tube and centrifuged at 1,000 rpm for 2 min at 4°C. Following centrifugation, the supernatant was removed, and organoid pellets were resuspended in a Cryostor CS10 cryopreservation medium (STEMCELL Technologies) at approximately 500–1,000 organoids/mL before being transferred to a cryovial. Cryovials were stored in a cryogenic freezing container for 24 h at −80°C and subsequently transferred to liquid nitrogen for long-term storage.

### Hematoxylin and eosin staining and immunohistochemical staining

The culture medium was removed from organoids cultured for 8 days, and the organoids were resuspended in 4% paraformaldehyde (Solarbio, Beijing, China) and incubated overnight at 4°C. The fixed sheep intestinal organoids were then embedded in 3% agarose. Hematoxylin and eosin (H&E) staining and immunohistochemical staining were performed on 5 µm sections of paraffin-embedded organoids. Paraffin-embedded organoid sections were dewaxed, and antigen retrieval was performed by pretreating them in boiling citrate buffer (Solarbio). Subsequently, slides were incubated in a blocking buffer consisting of 0.1% Triton X-100 (Sigma-Aldrich) and 5% BSA (Sigma-Aldrich) in PBS for 1 h at room temperature. Slides were incubated overnight at 4°C with the following primary antibodies: anti-CHGA (Huaan Biotechnology, Hangzhou, China) and anti-KI67 (Proteintech, Wuhan, China). After washing, the slides were incubated with antimouse and antirabbit secondary antibodies coupled to Alexa Fluor-488 (ABclonal, Wuhan, China) or Alexa Fluor-594 (ABclonal) for 1 h at room temperature. Diamidino-2-phenylindole (DAPI) (Biosharp, Anhui, China) was used to counterstain the DNA. All images were obtained with a confocal laser-scanning microscope (Zeiss, Oberkochen, Germany).

### Construction of *in vitro* inflammatory models

First, the colon organoids were cultured *in vitro* for 6 days. Then, the organoids were treated with either dextran sulfate sodium (DSS) (0.05% or 0.1%) for 24 h (33), TNF-α (10, 25, 100, and 150 ng/mL) for 48 h (34, 35), or lipopolysaccharide (LPS) (50, 100, and 200 µg/mL) for 72 h (36, 37). By systematically evaluating organoid growth status, apoptosis levels, and inflammatory factor expression, the optimal concentration that induces a moderate inflammatory response is identified. After the experiment, organoids were collected for subsequent analysis. The surface area of intestinal organoids was quantified using established methods (38).

### Assessment of apoptosis in intestinal organoids

Cell death in organoids (day 8) following 48 h of TNF-α treatment was evaluated using a live/dead staining assay. Matrigel was disrupted mechanically with a pipette tip, and organoids were transferred to an Eppendorf tube. Organoids were incubated with 2 μM calcein acetoxymethyl ester and 4 μM propidium iodide (PI) to assess cell viability (Beyotime Biotechnology). After 1 h of incubation in the dark, confocal microscopy was performed on a laser scanning microscope (Zeiss) (39).

### Preparation of the cell-free supernatant from *Faecalibacterium duncaniae*

*F. duncaniae* was obtained from the German Collection of Microorganisms and Cell Cultures. *F. duncaniae* was cultured in modified reinforced clostridium medium, containing 10.0 g/L glucose, 1.0 g/L soluble starch, 10.0 g/L tryptone, 3.0 g/L yeast extract, 10.0 g/L beef extract powder, 0.001 g/L resazurin, 5.0 g/L sodium chloride, 0.5 g/L L-cysteine hydrochloride, and 3.0 g/L sodium acetate, in an anaerobic workstation (N_2_:H₂:CO_2_ = 8:1:1) at 37°C. The cultures were terminated during the exponential growth phase, and the culture supernatants and bacterial pellets were separated by centrifugation at 12,000 rpm for 3 min at 4°C. The supernatants were filtered through a 0.22 μm membrane and stored at −20°C for future use.

### Treatment of intestinal organoids

Inflammatory intestinal organoids were pretreated with cell-free supernatant (CFS) at concentrations of 1%, 3%, and 5% for 24 h. The organoids were divided into five experimental groups (the organoids used in this experiment were fourth-passage organoids): control (CON, organoid culture medium), TNF-α (100 ng/mL, 48 h), 1% CFS + TNF-α, 3% CFS + TNF-α, and 5% CFS + TNF-α. All organoids were initially cultured in standard organoid medium from day 0. For the TNF-α group, organoids were exposed to TNF-α on day 6 for 48 h to induce inflammatory injury. For the CFS + TNF-α groups, organoids were pretreated with the indicated CFS concentrations on day 5 for 24 h prior to TNF-α stimulation (100 ng/mL, 48 h) (40). CFS concentrations were selected based on previous studies (41). To further assess the protective effects of the bacterial supernatant, organoids were also allocated to five groups: CON, TNF-α, 5 mM sodium butyrate (NaB) + TNF-α (positive control), 1% bacterial culture medium (BCM) + TNF-α (BCM, negative control), and 1% CFS + TNF-α. For the butyrate and BCM groups, organoids were pretreated for 24 h before TNF-α exposure. The butyrate concentration was chosen according to prior studies demonstrating its safety and physiological relevance for intestinal epithelial cells (42).

### Real-time qPCR

Total RNA was extracted from intestinal organoids (day 8) by the PaPure Total RNA Micro Kit (Magen, Guangzhou, China). The Evo M-MLV RT Reagent Kit (Accurate Biology, Hunan, China) was used to synthesize cDNA. Target gene fragments were amplified using SYBR Premix (Accurate Biology) and quantified on a QuantStudio 7 Flex system (Applied Biosystems). Gene mRNA expression analysis was performed using the 2−ΔΔCT method, with *POLR2G* as the housekeeping gene. The primers used in this study are listed in Table 1.

**TABLE 1:** Primers of real-time fluorescence quantitative PCR

Gene	Forward primer	Reverse primer
POLR2G	ACTTCGGCCCTAACTTGCTC	TGGTGGTGACGGCAATTACA
IL-1α	GTCCATACATGACGGCTGCT	TGTCTCAGGCATCTCCTTATGC
IL-6	TTCGGATGAAGTAGCTGCGG	AAGGACGTTCAAGCCGCATA
IL-8	AGGCATTTTTGAAGAGAGCTGAG	CCAGACCCACACAGTACTCAA
TNF-α	CTGGTTCAGACACTCAGGTCATC	ATGAGGTAAAGCCCGTCAGTG
MUC2	ACCTTCGACGGGCTCTACTA	TGTCGACGTAGATGCCGAAG
LGR5	TTGCAGGCAACCCTTCTCTT	GGCGCCATTCAAAGTCAGTG
FABP1	AGATCAAGGCGGTGGTTCAG	CCCTTCGTCATGGTACTGGT
GCG	GCCGATGGCTCTTTCTCTGA	TGACACACTTACTTCCTGTCAGT
ANKH	ACGTCATAGCTCAGGTCGTTTT	CCAGAGGCCACCAGAAACTC
ZO-1	CCAGACACTCTCCACAGCAG	TACACAGGCTTTGGCTCTGG
Claudin	GGCTTCATCCTGGCGTTTCT	GCTTGCAGAGTGCTGTTCAGA
Occludin	TACCACTCCTCCTCCGTAGC	AGATGAGTCAATCTGCGG

### Detection of epithelial proliferation in intestinal organoids

Intestinal organoids (day 8) in each well were incubated with 200 μL of 25 μM EdU (Beyotime Biotechnology) medium for 2 h, and the culture medium was then discarded. The organoids were fixed with paraformaldehyde for 1 h at 4°C and permeabilized with 0.3% Triton X-100 for 15 min; Hoechst 33342 was used to stain nuclei. Organoids were then detected with a confocal microscope, and the mean density of EdU cells was analyzed using ImageJ software. The percentage of EdU-positive cells was calculated as the number of EdU-positive cells divided by the total number of cells (DAPI), and values for each group were normalized to the mean of the control group.

### Paracellular permeability measurement in intestinal organoids

Based on previous studies (43), epithelial barrier function was assessed using a 4 kDa fluorescein isothiocyanate–dextran flux assay (4 kDa FITC-dextran [FD4]; Yeasen, Shanghai, China). A 1 mg/mL solution was prepared in nuclease-free water. After exposure, paracellular permeability was determined by co-incubating organoids (day 8) with FD4 at 37°C for 4 h. Confocal microscopy (Leica Microsystems GmbH, DE) and Image J software were used to assess FD4 permeation from the basal to luminal side of the organoids.

### Immunofluorescence staining and imaging of junctional proteins

Colon organoids were cultured in Matrigel until day 8. The fixation media were aspirated in the wells, and the organoids were washed thoroughly with cold PBS and incubated in neutrally buffered 4% paraformaldehyde (Sigma-Aldrich) for 1 h at room temperature. Subsequently, the organoids were permeabilized in PBS containing 0.1% Triton X-100 (vol/vol) (Sigma-Aldrich) for 15 min at room temperature. The blocking step was performed using 5% BSA (Sigma-Aldrich) in PBS for 1 h at room temperature. The organoids were thoroughly rinsed with PBS and incubated overnight at 4°C with the appropriate primary antibodies, such as anti-ZO-1 (ABclonal) and antioccludin (ABclonal). The marker gene expressions were detected by incubating the samples with corresponding secondary antibodies coupled to AlexaFluor 488 (ABclonal) for 1 h at room temperature. Samples were counterstained with DAPI and mounted on glass slides using Antifade Mounting Medium for Fluorescence (Biosharp) mounting medium. Confocal images were acquired using a confocal microscope (Leica Microsystems GmbH). Images were then analyzed using the ImageJ software. Protein mean gray values of each organoid were normalized to the corresponding DAPI intensity and then to the control group mean.

### Western blot analysis

Proteins were extracted from colon organoids (day 8) with RIPA lysis buffer (Boerfu Biotechnology, Wuhan, China) supplemented with the 1% protease inhibitor PMSF (Biosharp) and phosphatase inhibitor (Meilunbio, Liaoning, China). Protein concentration was measured using a BCA kit (Beyotime Biotechnology, Wuhan, China). The samples were subjected to sodium dodecyl sulfate‒polyacrylamide gel electrophoresis (SDS‒PAGE, EpiZyme), transferred to PVDF membranes (Millipore, Shanghai, China), blocked with 5% skim milk (Biosharp), and incubated at 4°C overnight with the primary antibodies against ZO-1 (1:2,500; ABclonal) and occludin (1:1,000; ABclonal). Then, the corresponding HRP-conjugated secondary antibodies (1:5,000; ABclonal) were applied. Protein bands were visualized by the chemiluminescence imaging system (Tanon, Shanghai, China). The relative expression levels of the target protein were quantified by densitometry, normalized to β-actin, and subsequently expressed relative to the mean value of the control group.

### RNA-seq

To investigate the regulatory mechanism of *F. duncaniae* on inflammatory organoids, cell samples from the CON group, TNF-α group, and 1% CFS + TNF-α group (day 8) were collected. RNA extraction was performed as described above. High-quality RNA samples (OD260/280 = 1.8–2.2, OD260/230 ≥ 2.0, RIN ≥ 6.5, 28S:18S ≥ 1.0, >10 µg) were used to construct a sequencing library. RNA-seq libraries were prepared from 1 μg of total RNA using standard procedures, including mRNA isolation via poly(A) selection, fragmentation, cDNA synthesis, end repair, A-base addition, and ligation of indexed adapters. Libraries were size-selected for 200–300 bp fragments and PCR-amplified for 15 cycles. After quantification, the final libraries were sequenced on the DNBSEQ-T7 platform (Shanghai BIOZERON Biotech. Co., Ltd.). The raw paired-end reads were trimmed and quality controlled by Trimmomatic (44) with parameters (SLIDINGWINDOW:4:15 MINLEN:75) (version 0.36) (27). Low-quality reads, adapter sequences, and short sequences ( <75 bp) were removed. Clean reads were aligned to the reference genome using Hisat2 with default parameters, and quality was assessed with Qualimap (version 2.2.1). The proportion of rRNA was evaluated using Bowtie2, and gene reads were counted with featureCount.

### Differential expression analysis and functional enrichment

A post hoc Dunn’s test was applied to evaluate differences in Kyoto Encyclopedia of Genes and Genomes (KEGG) annotation abundances (45) among the three sample types: CON, TNF-α, 1% CFS + TNF-α (the 1% CFS + TNF-α group is hereafter referred to as the CFS group), with *P* values adjusted using the Bonferroni method and statistical significance defined as *P*_adj_ < 0.05. Mean transcripts-per-million (TPM) values per sample were used to calculate average functional abundance, with a minimum abundance threshold of TPM ≥0.5 across all samples. To integrate KEGG pathway enrichment with gene-level functional profiles, core enrichment gene sets from gene set enrichment analysis results were extracted and annotated using Entrez and symbol identifiers. Genes with significant abundance differences (Dunn’s test, *P*_adj_ < 0.05) and transcriptional relevance (TPM ≥0.5 in at least one sample type) were retained. These gene sets were merged with KEGG metadata (description, NES, *P* value) and average TPM values across the three sample types to generate pathway–gene association tables for each comparison group (1% CFS + TNF-α vs TNF-α and CON vs TNF-α). The specific gene information is provided in Data S2.

### Constructing the protein–protein interaction network

To explore the interactions among differentially expressed genes between the TNF-α group and the 1% CFS + TNF-α group, a protein–protein interaction (PPI) network was constructed using the STRING database (46) (https://string-db.org/), setting the composite score of >0.4 as the cutoff in order to further understand the interaction between the corresponding differential genes and screen the significant molecules (47). The TSV document was downloaded and submitted to the Cytoscape software to generate a visualized network.

### Establishment of core modules and core genes

The MCODE plugin in Cytoscape software with default thresholds (K-core = 2, node score cutoff = 0.2, degree cutoff = 2, maximum depth = 100) was used to screen important functional modules in the PPI network (46). The cytoHubba plugin was used to screen key nodes in the PPI network (47). The cytoHubba plugin uses 12 topological analysis strategies to predict and evaluate important nodes, and nodes that score in the top 10 in more than five algorithms are considered core genes (48, 49).

### Statistical analysis

The results are expressed as means ± SDs. One-way ANOVA was employed to determine significant differences among multiple groups, and the *t*-test was employed to assess differences between the two groups. *P < 0.05, **P < 0.01, ***P < 0.001, and ****P < 0.0001. Data were combined from at least three independent experiments unless otherwise stated. GraphPad Prism 7 software was used for statistical analysis.

## RESULTS

### *Faecalibacterium duncaniae* is associated with gut health status in dairy calves

**Fig 1:**
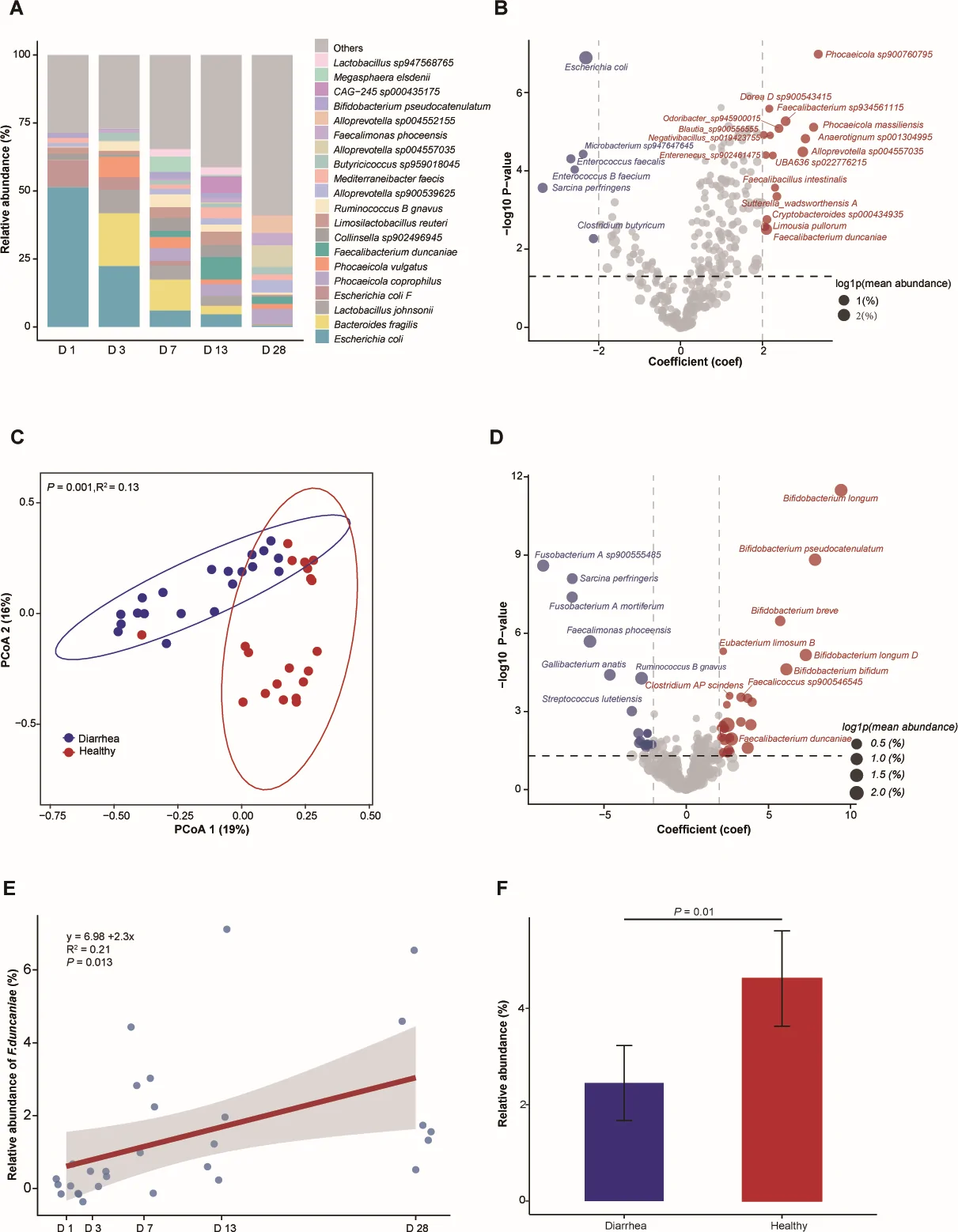
Microbial community dynamics and *Faecalibacterium duncaniae* abundance in early cultivation stages. (**A**) Distribution of microbiome community types at different time points. (**B**) Time-series analysis of species abundance based on MaAsLin2. (**C**) PCoA based on Bray−Curtis distance, illustrating the variances in microbial structure. (**D**) MaAsLin2-based analysis of differential gut microbiota between healthy and diarrheic calves. (**E**) The abundance dynamics of *Faecalibacterium duncaniae* during the early cultivation stage over time. (**F**) Abundance variation of *Faecalibacterium duncaniae* in the healthy and diarrheic groups.

To identify microbial taxa associated with intestinal health during early life, we first characterized the longitudinal development of the rectal microbiota in neonatal calves. Community profiling revealed marked temporal shifts in microbial composition during the first month after birth (Fig. 1A). To systematically identify taxa associated with early-life microbial succession, we applied the multivariable statistical framework MaAsLin2, using sampling time as a continuous variable. Several taxa displayed consistent developmental trajectories (Fig. 1B), including species that progressively increased during early growth (e.g., *Phocaeicola* sp900760795, *Faecalibacillus intestinalis*, and *Faecalibacterium duncaniae*) as well as taxa that declined over time (*Escherichia coli*, *Enterococcus faecalis*, and *Clostridium butyricum*). Additionally, significant differences were observed in the overall structure of the fecal microbiota between the diarrhea and healthy groups (Fig. 1C). Differential abundance analysis was also performed using MaAsLin2, identifying multiple bacterial species (*Bifidobacterium longum*, *Fusobacterium A mortiferum*, and *Faecalibacterium duncaniae*) associated with health status (Fig. 1D). Notably, comparison between the longitudinal developmental analysis and the disease-associated differential analysis revealed that *F. duncaniae* was the only species that both increased during normal early-life microbiota development (Fig. 1E) and was significantly enriched in healthy calves relative to diarrheic individuals (Fig. 1F). Together, these findings suggest that depletion of *F. duncaniae* may represent a deviation from the normal developmental trajectory of the calf gut microbiota and highlight this species as a candidate microbial indicator of intestinal health during early life.

### Establishment of a ruminant colonic organoid model to investigate epithelial injury and repair

Although the association between *F. duncaniae* and calf gut health was evident, determining its direct effects on intestinal epithelial function requires a controlled experimental system that allows mechanistic interrogation. To this end, we established a three-dimensional colonic organoid model derived from ruminant intestinal epithelium as a physiologically relevant *in vitro* platform to study epithelial injury and repair processes.

**Fig 2:**
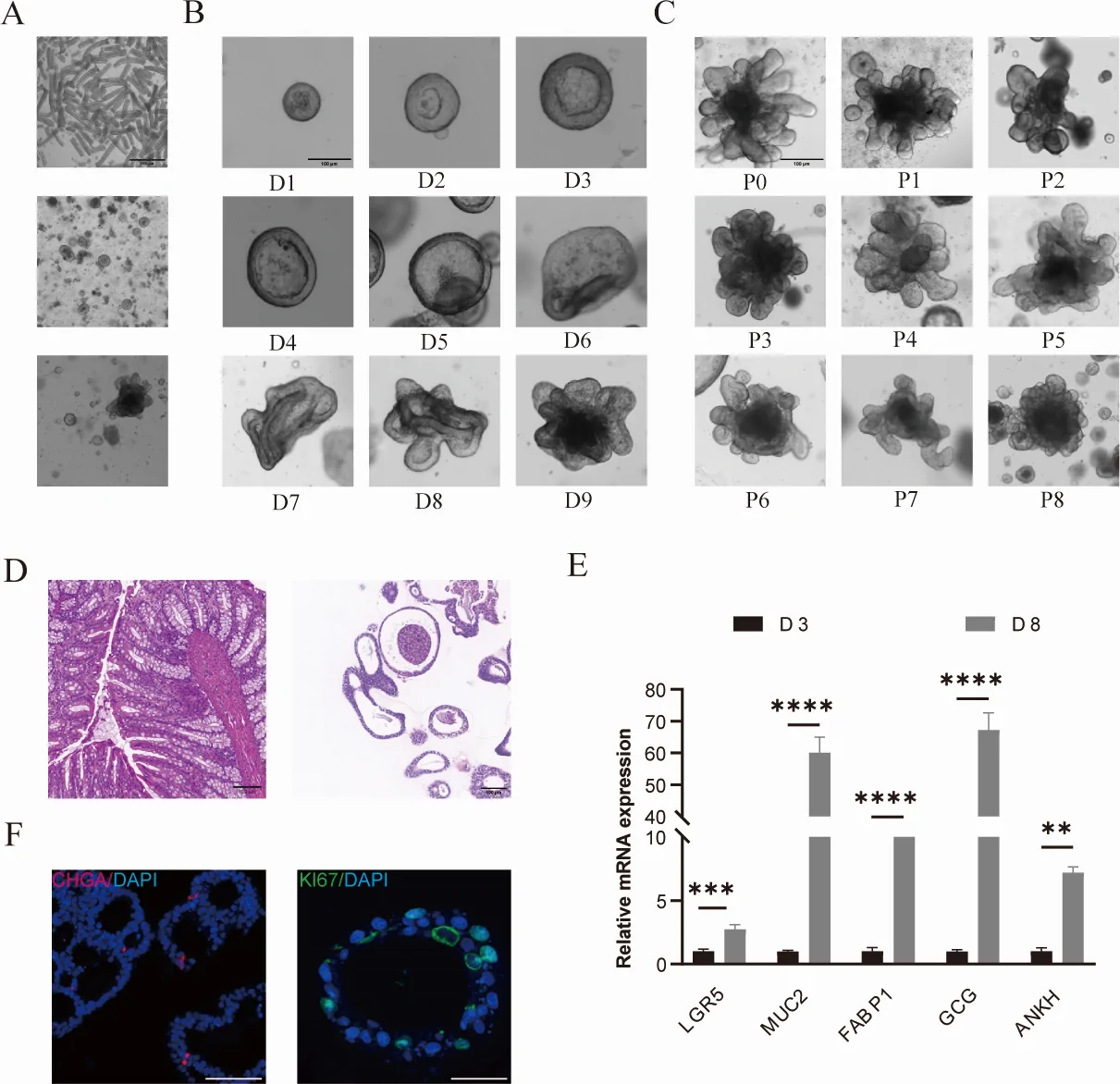
Establishment of sheep colon organoids. (**A**) Sheep colon crypts (top), initial morphology of organoids (middle), organoids’ mature morphology (bottom). Scale bar: 100 μm. (**B**) Images of the same organoid at the indicated days. Scale bar: 100 μm. (**C**) Representative images of sheep colon organoids at different passages. The P4 image and the 0 h image (see Fig. 4) (CON group) were derived from the same original image and are shown here solely to represent the morphology of fourth-passage organoids. Scale bar: 100 μm. (**D**) Representative images of intestinal tissue and organoid HE sections (day 8). Scale bars: 100 μm. (**E**) RT-qPCR was used to assess marker gene expression in the organoid at the indicated time points (*n* = 3). (**F**) Representative images of immunofluorescence staining show intestinal endocrine cells (CHGA, red) and amplified cells (KI67, green) in the organoid. DNA was stained with DAPI (blue). Scale bars: 50 μm. Data are presented as mean ± SD. Differences between the two groups were evaluated using Student’s *t*-test. **P < 0.01, ***P < 0.001, ****P < 0.0001.

Crypts were isolated from the colonic tissues to establish intestinal organoids *in vitro* (Fig. 2A). Following single-crypt tracking, initial organoid cultures progressed from elongated, intact crypts to condensed spheroids. From days 2 to 6, colonic organoids possessed an enclosed central lumen and began to bud into a new region with a crypt-like structure (Fig. 2B). Branching continued between days 7 and 9, with increasing bud numbers and a pronounced luminal architecture, accompanied by progressive growth in size. Organoids matured when their diameters reached 100–200 μm, displaying crypt–villus-like structures and containing stem and differentiated cell compartments (50). They could stably be passaged at least eight times without significant morphological changes (Fig. 2C). The results showed that colonic crypt-derived intestinal organoids possess rapid proliferative capacity and can be sustained long-term while retaining their capacity to recapitulate intestinal function.

H&E staining showed that ovine colonic organoids formed a central lumen lined by a single layer of columnar epithelial cells, with numerous shed cells present, reflecting morphological similarity to native intestinal tissues (Fig. 2D). We evaluated the expression of key epithelial markers, including stem cells (*LGR５*), goblet cells (*MUC２*), enteroendocrine cells (*GCG*), and absorptive enterocytes (*FABP１*and *ANKH*), in the RNA of colon organoids at early and late culture stages (Fig. 2E). We found that the expression levels of these marker genes increased with prolonged culture time, indicating that organoids gradually developed multiple intestinal epithelial lineages during extended culture. In addition, immunofluorescence staining confirmed the presence of the enteroendocrine cell marker (CHGA) and the proliferation marker (KI6７) (Fig. 2F). Together, these results suggested that colon organoids accurately captured the key cellular lineage features of intestinal tissue at both morphological and molecular levels.

### TNF-α induces reproducible inflammatory epithelial injury in ruminant colonic organoids

To functionally evaluate the effects of *F. duncaniae* under conditions relevant to calf intestinal inflammation, a reproducible epithelial injury model was established in ruminant colonic organoids. Organoids were exposed to commonly used inflammatory stimuli, including DSS, LPS, and TNF-α. DSS is a chemical agent that can disrupt the intestinal epithelium (51). At concentrations as low as 0.05%, it systematically disrupted the organoid structure, causing morphological changes and basement membrane damage (Fig. S1A and B). Additionally, later stages of treatment led to the drift and disintegration of some organoids. Due to its high cytotoxicity and limited ability to induce expression of inflammatory factors (Fig. S1C), it was unsuitable for constructing subsequent models (52). LPS activates the immune response through Toll-like receptor 4 (TLR4) (53). The administration of 100 μg/mL LPS significantly activates the expression of *IL-8*, *IL-6*, *TNF-α*, and *IL-1α* (Fig. S1D). It does not cause significant phenotypic changes in organoids compared to other concentration groups (Fig. S1E and F).

**Fig 3:**
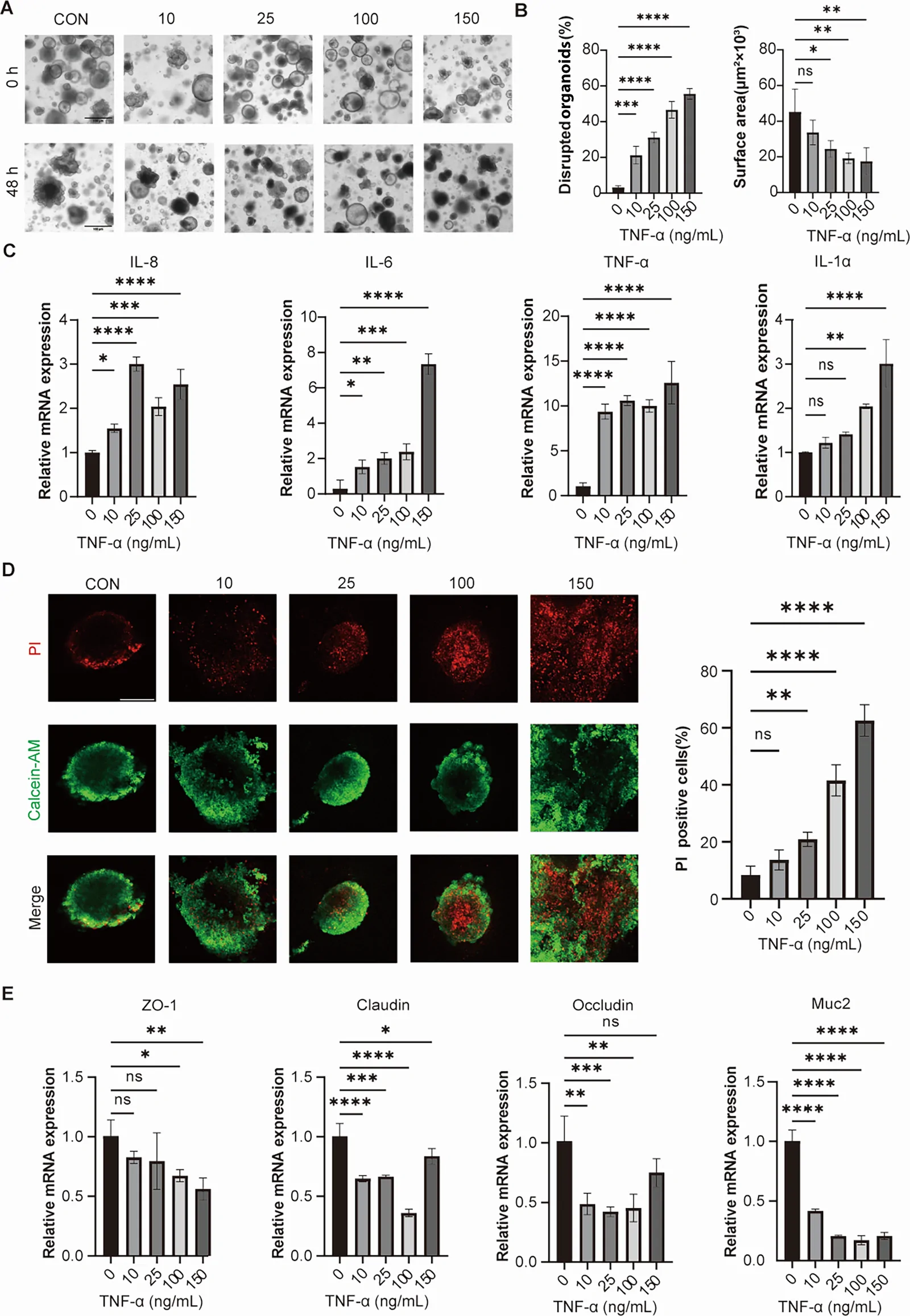
Construction of an *in vitro* organoid inflammation model. (**A**) Organoids (day 6) were treated with different concentrations of TNF-α for 48 h (day 8). Scale bars: 100 μm. (**B**) The morphology of organoids after TNF-α treatment was assessed using optical microscopy (*n* = 3). (**C**) Inflammatory cytokine secretion levels after TNF-α treatment (*n* = 3). (**D**) Calcein acetoxymethyl ester (Calcein-AM)/PI fluorescence images of colonic organoids. Scale bars: 50 μm (*n* = 3). (**E**) qRT-PCR was used to evaluate the expression of TNF-α-stimulated intestinal epithelial barrier-related genes in colon organoids (*n* = 3). Data are presented as mean ± SD. Data were analyzed using one-way ANOVA followed by Dunnett’s multiple comparison test, with comparisons made against the 0 ng/mL group. ns, no significant difference. *P < 0.05, **P < 0.01, ***P < 0.001, ****P < 0.0001.

In contrast, different concentrations of TNF-α can induce concentration-dependent damage in organoids (Fig. 3A and B). Compared with the control group, the secretion levels of the inflammatory cytokines *TNF-α*, *IL-6*, *IL-1α*, and *IL-8* were significantly increased (Fig. 3C). Live/dead analysis of the organoids further confirmed that as TNF-α concentration increased from low to high, the proportion of PI-positive cells gradually increased, accompanied by damage to epithelial structures (Fig. 3D). At 100 ng/mL, the organoids exhibited moderate apoptosis and partial structural changes, without complete loss of functionality, making them suitable for simulating inflammatory responses under physiological conditions. At 150 ng/mL, more than 60% of the organoids exhibited irreversible damage and decreased activity, indicating that this concentration is too high and unsuitable for subsequent experiments.

TNF-α promotes epithelial barrier damage by disrupting tight junctions (54). This effect is more pronounced in the high-concentration groups (especially at 100 ng/mL) (Fig. 3E). Based on the observed inflammatory phenotype and cell apoptosis, we selected 100 ng/mL TNF-α as the optimal concentration to induce inflammation in organoids in subsequent experiments.

### *Faecalibacterium duncaniae*-derived metabolites attenuate inflammatory epithelial injury

Based on the observed association between *F. duncaniae* and calf gut health, we further investigated whether *F. duncaniae* could exert protective effects on intestinal epithelial cells under inflammatory stress *in vitro*. However, as *F. duncaniae* is an obligate anaerobe, maintaining viable bacterial cells under conventional organoid culture conditions presents technical limitations. Notably, *F. duncaniae* is a well-recognized butyrate-producing bacterium, and its secreted metabolites have been reported to play important roles in maintaining intestinal barrier integrity and regulating inflammatory responses. Therefore, we used the CFS of *F. duncaniae* to evaluate the functional effects of its secreted metabolites. Specifically, colonic organoids were treated with CFS both prior to and during TNF-α-induced inflammatory challenge to assess its potential protective effects against inflammatory injury.

**Fig 4:**
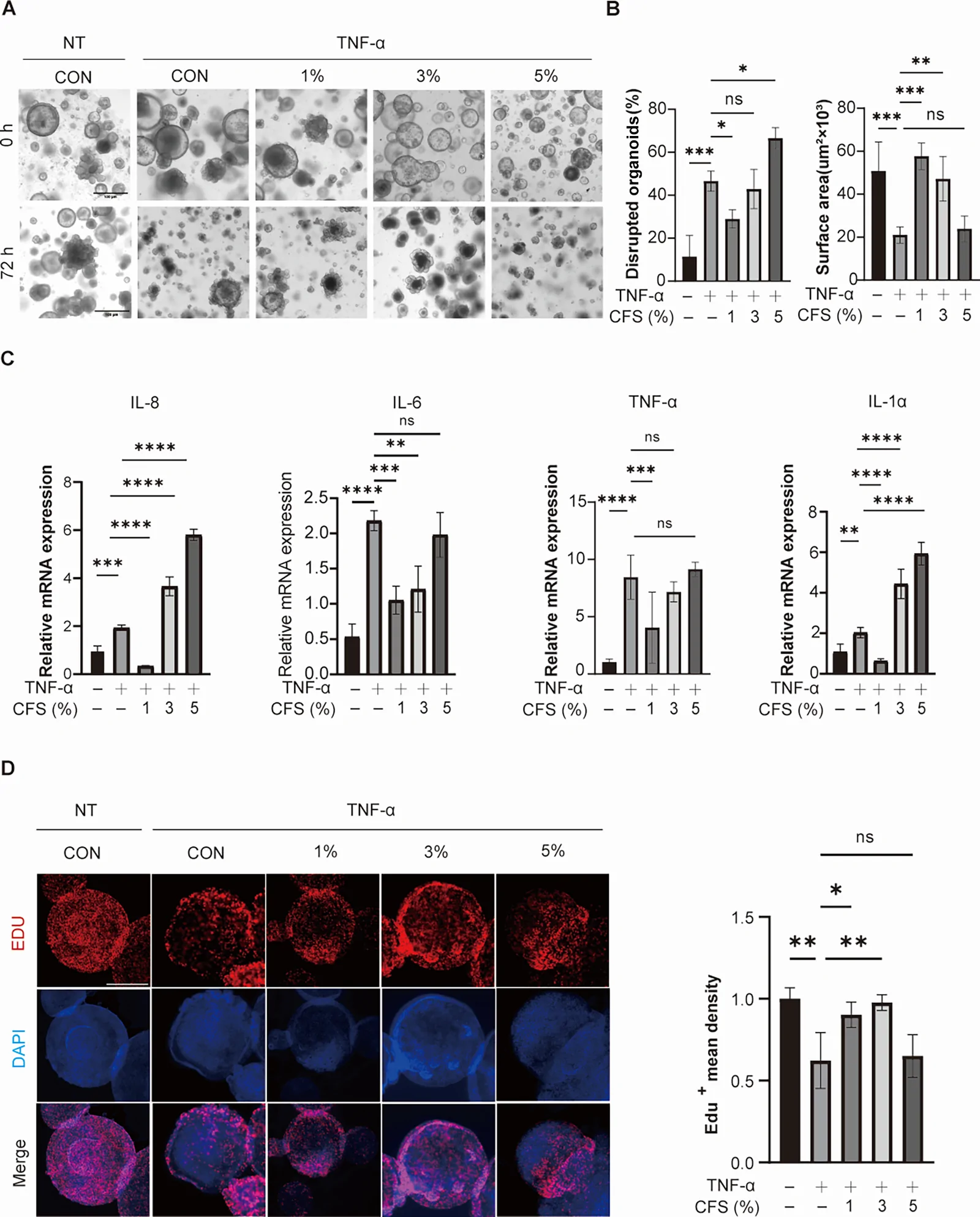
Reduction of inflammatory response and promotion of epithelial cell proliferation under co-incubation of CFS and TNF-α. (**A**) Organoids (day 5) were treated with CFS alone for 24 h and co-incubated with TNF-α (100 ng/mL) for another 48 h (day 8). Scale bars: 100 μm. (**B**) The morphology of organoids was evaluated by optical microscopy (*n* = 3). (**C**) qRT-PCR was used to evaluate the expression of inflammation-related genes in colonic organoids under co-incubation conditions (*n* = 3). (**D**) Confocal images of nuclear staining (blue) and EdU staining (red) in organoids. Scale bars: 50 μm. The proportion of EdU-positive cells relative to total cells (DAPI-stained nuclei) was calculated for each organoid, and values were normalized to the mean of the control group (*n* = 3). Data are presented as mean ± SD. Data were analyzed using one-way ANOVA followed by Dunnett’s multiple comparison test, with comparisons made against the TNF-α group. ns, no significant difference. *P < 0.05, **P < 0.01, ***P < 0.001, ****P < 0.0001.

Compared with TNF-α treatment, CFS-treated organoids significantly increased organoid surface area and reduced the number of damaged organoids (Fig. 4A and B). In parallel, CFS treatment markedly reduced the expression of inflammation-related genes, including *TNF-α*, *IL-6*, *IL-1α*, and *IL-8* (Fig. 4C). EdU incorporation assays further revealed that CFS treatment restored epithelial proliferation suppressed by TNF-α exposure and reduced cell loss, which suggests the initiation of epithelial regeneration (Fig. 4D). These findings indicate that *F. duncaniae*-derived metabolites directly mitigate inflammatory epithelial injury and promote intestinal epithelial cell regeneration.

### *Faecalibacterium duncaniae*-derived metabolites preserve epithelial barrier integrity under inflammatory stress

**Fig 5:**
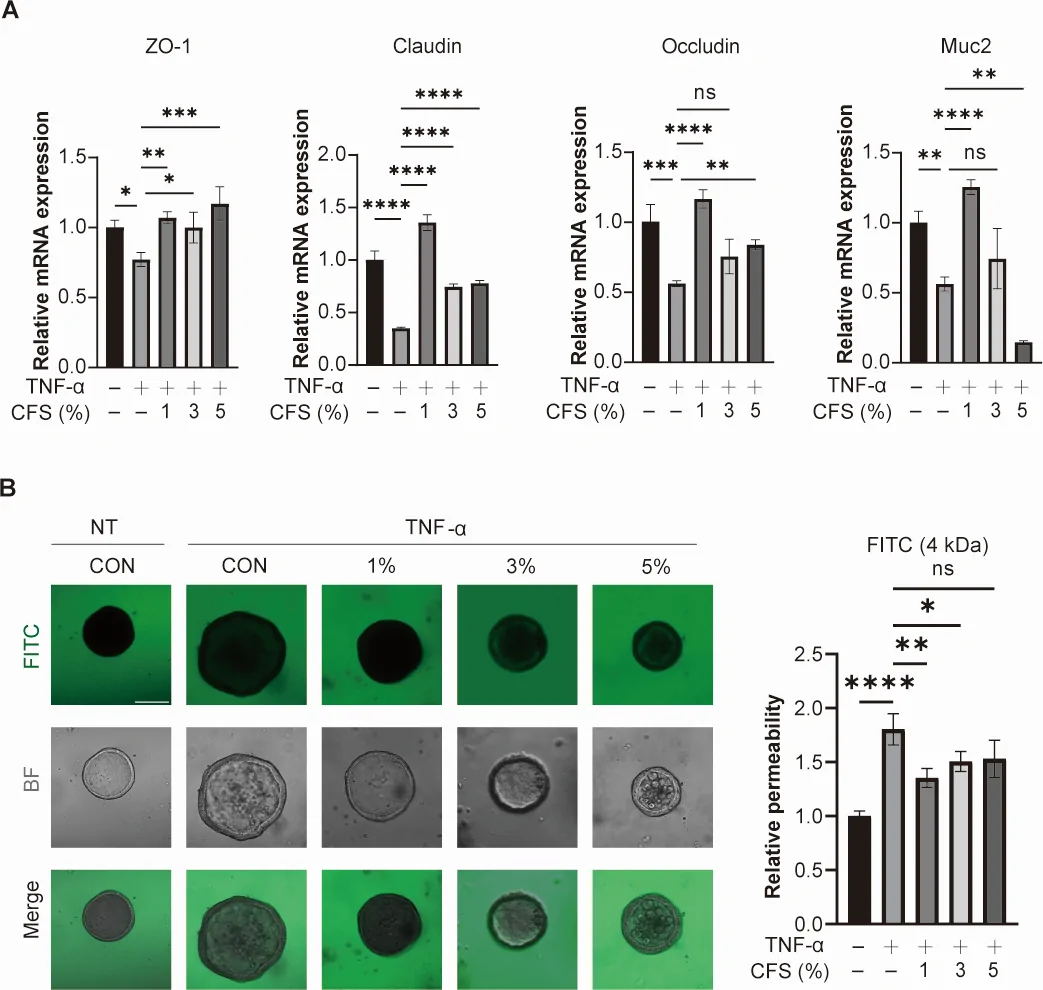
Improvement of epithelial barrier permeability by co-incubation of CFS and TNF-α. (**A**) qRT-PCR was used to evaluate the expression of intestinal epithelial barrier-related genes in colon organoids under co-incubation conditions (*n* = 3). (**B**) Changes in intestinal epithelial permeability of colonic organoids under co-incubation conditions. Scale bars: 50 μm (*n* = 3). Data are presented as mean ± SD. Data were analyzed using one-way ANOVA followed by Dunnett’s multiple comparison test, with comparisons made against the TNF-α group. ns, no significant difference. *P < 0.05, **P < 0.01, ***P < 0.001, ****P < 0.0001.

**Fig 6:**
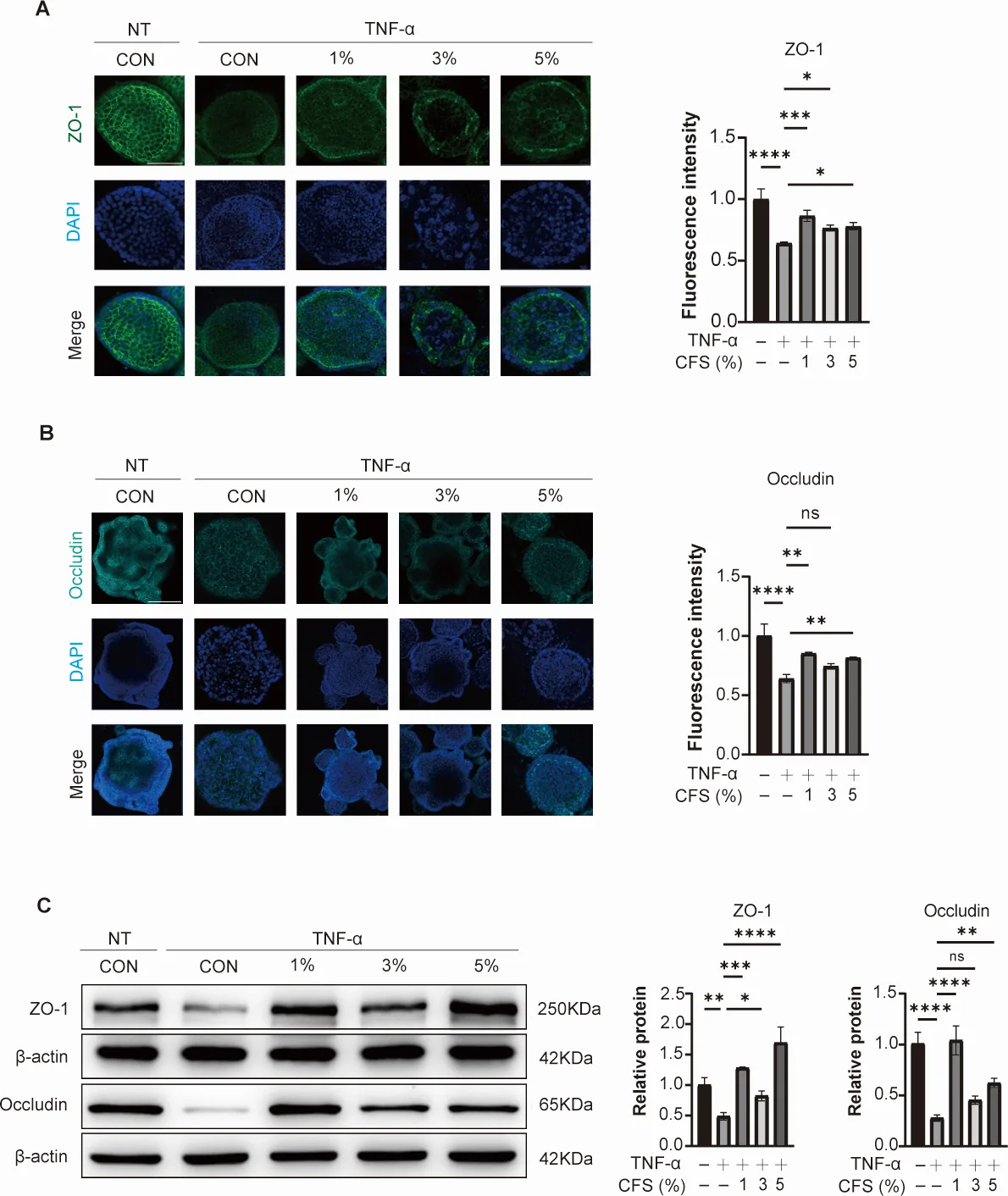
Improvement of epithelial barrier permeability by co-incubation of CFS and TNF-α. (**A**) Representative immunofluorescence staining and quantification of ZO-1 (green) in colonic organoids. Scale bars: 50 μm (*n* = 3). (**B**) Representative immunofluorescence staining and quantification of occludin (blue) in colonic organoids. Scale bars: 50 μm. Protein mean gray values of each organoid were normalized to the corresponding DAPI intensity and then to the control group mean (*n* = 3). (**C**) The protein expression and abundance of ZO-1 and occludin. Protein band intensities were normalized to β-actin and then to the mean of the control group (*n* = 3). Data are presented as mean ± SD. Data were analyzed using one-way ANOVA followed by Dunnett’s multiple comparison test, with comparisons made against the TNF-α group. ns, no significant difference. *P < 0.05, **P < 0.01, ***P < 0.001, ****P < 0.0001.

Given the central role of epithelial barrier dysfunction in calf intestinal inflammation and diarrhea, we further assessed the effects of *F. duncaniae*-derived metabolites on barrier-related outcomes. Compared with the TNF-α group, the CFS-treated organoids exhibited significant upregulation of tight junction protein gene expression (Fig. 5A). Permeability assays demonstrated that TNF-α treatment significantly increased paracellular macromolecular flux in intestinal organoids, whereas co-treatment with CFS partially reversed this effect, reducing epithelial permeability and indicating improved barrier integrity (Fig. 5B). Immunofluorescence (Fig. 6A and B) and Western blot (Fig. 6C) analyses confirmed that CFS improved the loss of ZO-1 and occludin. Collectively, these results demonstrate that *F. duncaniae*-derived metabolites protect epithelial barrier integrity under inflammatory conditions relevant to calf gut health.

### *Faecalibacterium duncaniae*-derived metabolites alleviate TNF-α-induced inflammation and intestinal barrier damage by reducing NF-κB activation and enhancing regulatory and repair pathways

**Fig 7:**
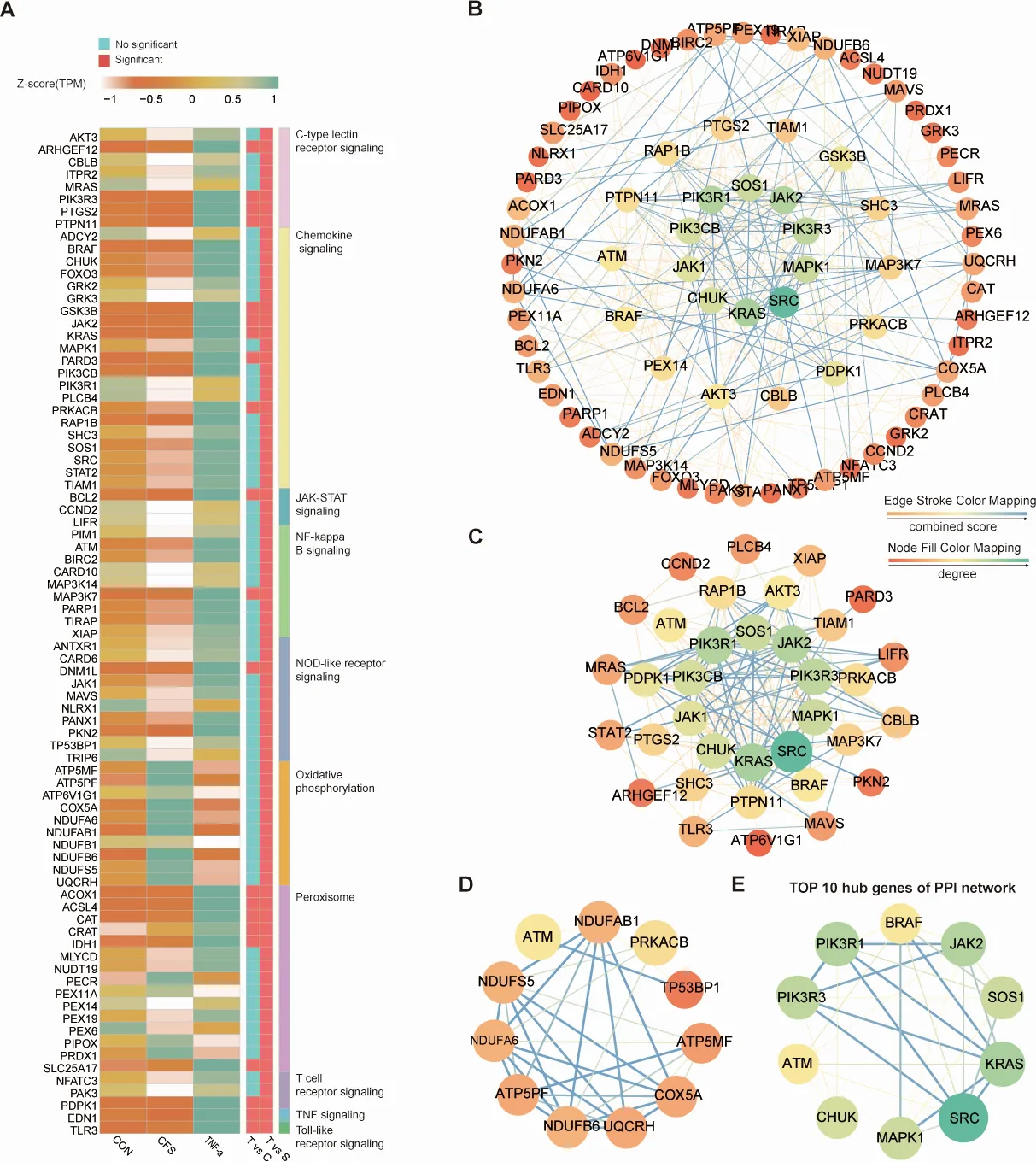
Through co-incubation of 1% CFS with inflammatory organoids, NF-κB activation is suppressed, and regulatory and repair pathways are enhanced. (**A**) Heatmap of differential pathway signaling and downstream signaling genes (C, CON; T, TNF-α; S, 1% CFS + TNF-α). Data are presented as normalized *Z*-scores. Statistical significance was determined using Dunn’s post hoc test. (**B**) PPI networks of the common differentially expressed genes. In the network, nodes represent proteins, and edges indicate interactions (TNF-α vs 1% CFS + TNF-α). (**C and D**) The two highest-scoring modules are arranged from top to bottom. (**E**) The PPI hub gene module.

To explore the mechanisms by which *F. duncaniae*-derived metabolites alleviate inflammation in organoids, RNA sequencing analysis was performed on organoids from the control, TNF-α, and 1% CFS + TNF-α groups. KEGG analysis showed that pathways including TNF, chemokine, Toll-like receptor, and NF-κB signaling were altered among the three groups (Fig. S2A). Within the NF-κB pathway, compared with the TNF-α group, the 1% CFS + TNF-α group showed downregulation of key signaling mediators such as *MAP3K7*, *MAP3K14*, and *TIRAP*. In addition, multiple genes involved in antiapoptotic activity and negative feedback regulation of inflammation, including *BIRC2* and *XIAP*, were also downregulated, suggesting a pronounced anti-inflammatory and survival response (Fig. 7A).

To further elucidate the regulatory network mediated by CFS, we constructed a PPI network based on the differentially expressed genes between the TNF-α group and the 1% CFS + TNF-α group. The resulting network consisted of 80 nodes and 296 interaction edges (Fig. 7B). MCODE analysis identified two functional modules within the network. The first module was mainly associated with NF-κB signaling, chemokine signaling, and other inflammation-related pathways, whereas the second module was primarily related to oxidative phosphorylation (Fig. 7C and D). Using the cytoHubba plugin for hub gene analysis, we identified nine key regulatory genes: *SRC*, *KRAS*, *SOS1*, *JAK2*, *BRAF*, *PIK3R1*, *ATM*, *CHUK*, and *MAPK1* (Fig. 7E). These genes primarily originated from the first module and play essential roles in immune regulation and inflammatory signaling.

### *Faecalibacterium duncaniae*-derived metabolites: butyrate attenuates TNF-α-induced inflammatory response and disruption of epithelial barrier integrity

**Fig 8:**
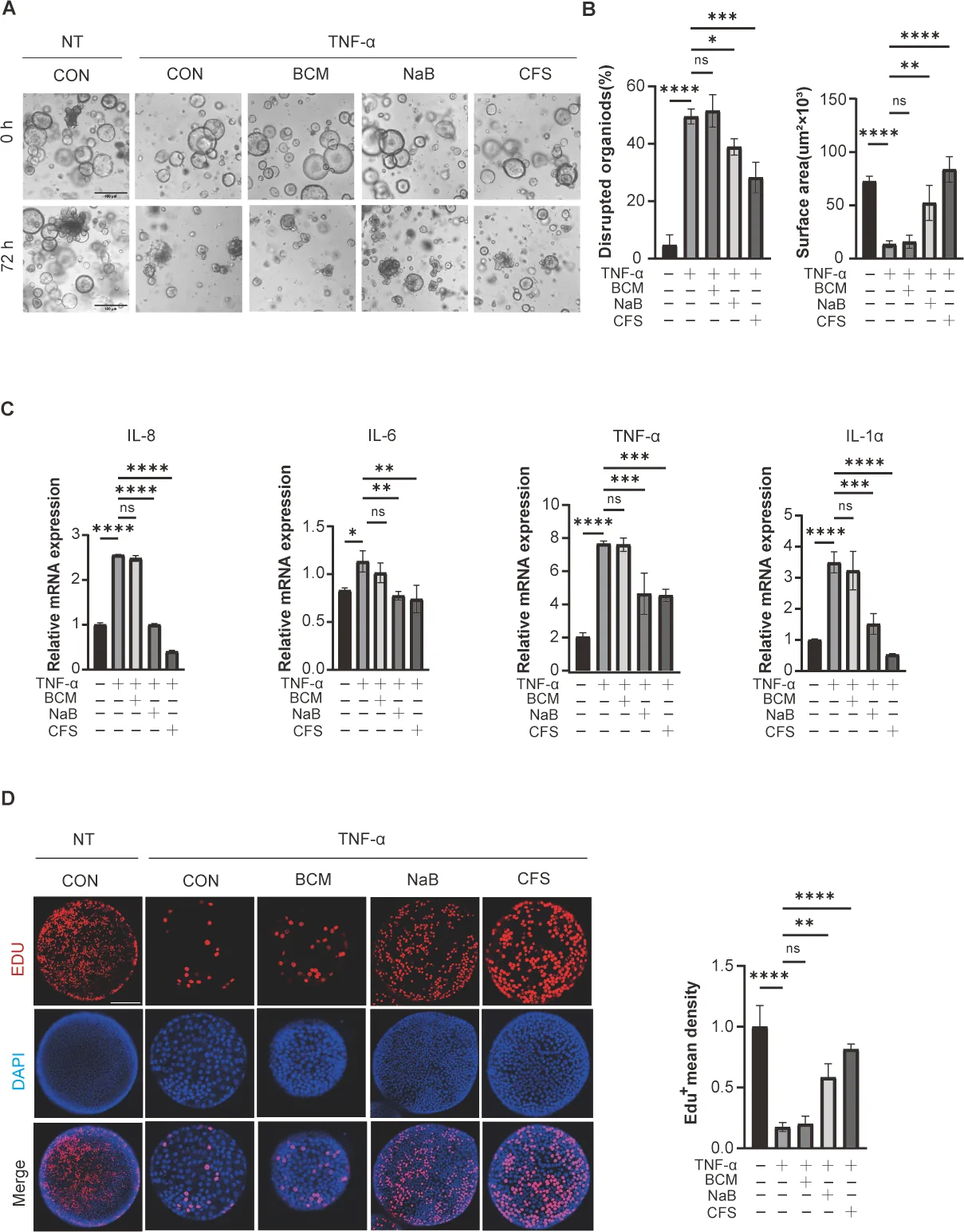
Butyrate attenuates TNF-α-induced organoid injury and promotes epithelial cell proliferation. (**A**) Organoids (day 5) were pretreated with 5 mM NaB, 1% BCM, or 1% CFS for 24 h, followed by co-incubation with TNF-α (100 ng/mL) for 48 h (day 8). Scale bars: 100 μm. (**B**) The morphology of organoids was evaluated by optical microscopy (*n* = 3). (**C**) qRT-PCR was used to evaluate the expression of inflammation-related genes in colonic organoids under co-incubation conditions (*n* = 3). (**D**) Confocal images of nuclear staining (blue) and EdU staining (red) in organoids. Scale bars: 50 μm. The proportion of EdU-positive cells relative to total cells (DAPI-stained nuclei) was calculated for each organoid, and values were normalized to the mean of the control group (*n* = 3). Data are presented as mean ± SD. Data were analyzed using one-way ANOVA followed by Dunnett’s multiple comparison test, with comparisons made against the TNF-α group. ns, no significant difference. *P < 0.05, **P < 0.01, ***P < 0.001, ****P < 0.0001.

**Fig 9:**
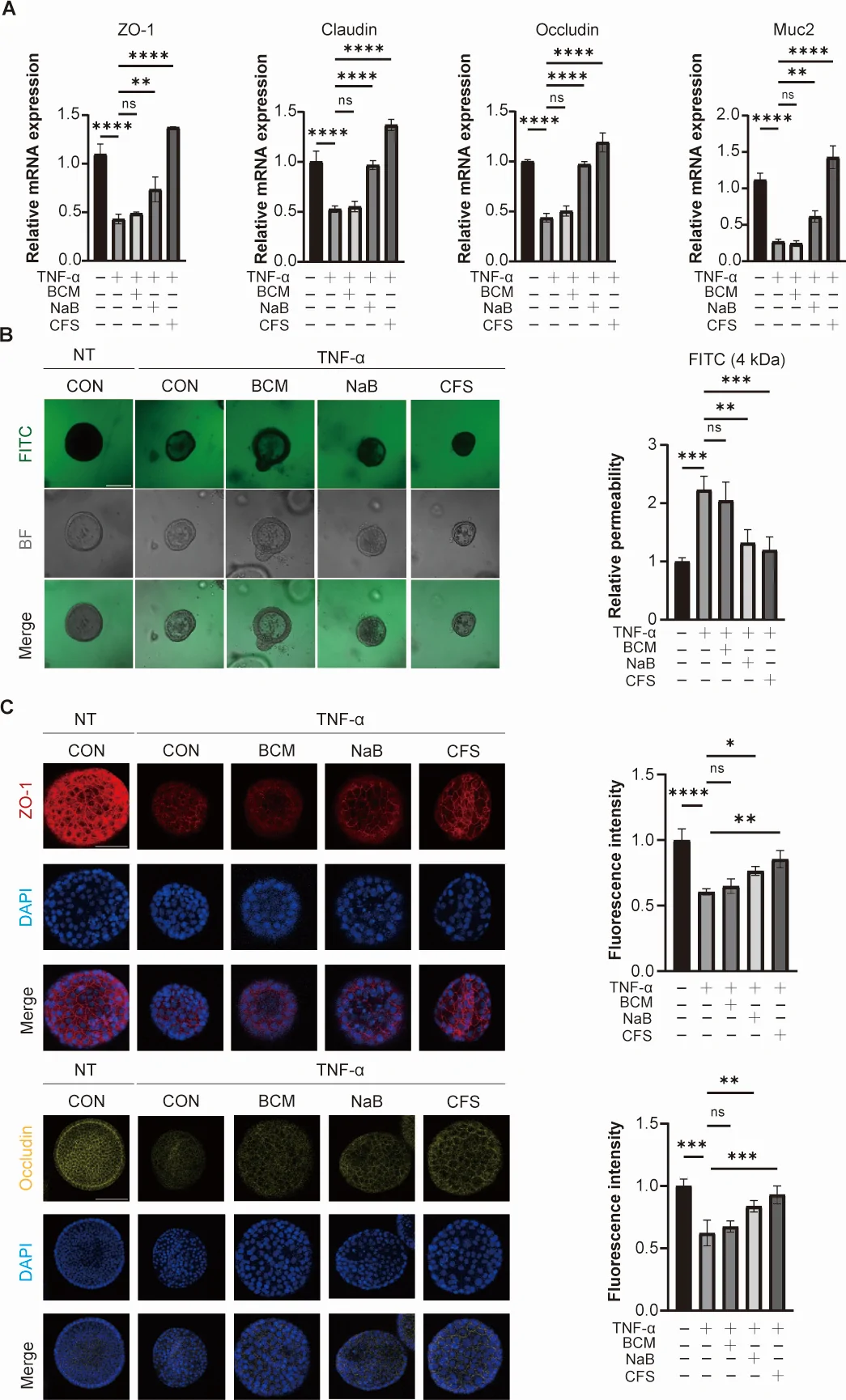
Butyrate attenuates epithelial barrier disruption. (**A**) qRT-PCR was used to evaluate the expression of intestinal epithelial barrier-related genes in colon organoids under co-incubation conditions (*n* = 3). (**B**) Changes in intestinal epithelial permeability of colonic organoids under co-incubation conditions. Scale bars: 50 μm (*n* = 3). (**C**) Representative immunofluorescence staining and quantification of ZO-1 (red) and Occludin (yellow) in colonic organoids. Scale bars: 50 μm. Protein mean gray values of each organoid were normalized to the corresponding DAPI intensity and then to the control group mean (n = 3). Representative immunofluorescence staining and quantification of occludin (yellow) in colonic organoids. Scale bars: 50 μm (*n* = 3). Data are presented as mean ± SD. Data were analyzed using one-way ANOVA followed by Dunnett’s multiple comparison test, with comparisons made against the TNF-α group. ns, no significant difference. *P < 0.05, **P < 0.01, ***P < 0.001, ****P < 0.0001.

SCFAs are the main metabolites of *F. duncaniae*, with butyrate serving as an important energy source for colonocytes (55). Butyrate can exert anti-inflammatory effects by maintaining epithelial barrier function and suppressing pro-inflammatory cytokine production (56). To further evaluate the protective effects of *F. duncaniae* metabolites, inflammatory organoids were pretreated with 5 mM NaB, 1% CFS (the optimal concentration in our model), or 1% BCM (as a control) (Fig. 8A). NaB treatment improved organoid morphology (Fig. 8B), alleviated TNF-α-induced inflammatory responses (Fig. 8C), partially restored epithelial proliferation (Fig. 8D) and barrier integrity (Fig. 9A and C), and reduced TNF-α-induced increases in paracellular permeability (Fig. 9B). In comparison, pretreatment with 1% CFS provided more pronounced protective effects than NaB, suggesting that additional active metabolites in CFS may contribute to anti-inflammatory and barrier-restorative functions. Additionally, the BCM group showed no significant improvement, indicating that the culture medium itself had little impact on inflammatory injury.

## DISCUSSION

In recent years, gut microbiota has emerged as a central focus in studies of intestinal health and disease prognosis. In the present study, we observed a consistent depletion of *F. duncaniae* in diarrheic dairy calves, suggesting that the loss of this commensal bacterium may be closely associated with early-life intestinal dysfunction (57). To move beyond correlative analysis and investigate its functional significance, we established a ruminant colonic organoid model to examine the protective effects of *F. duncaniae* metabolites on epithelial barrier integrity under inflammatory conditions.

We successfully generated stable, passagable ruminant colonic organoids that faithfully recapitulate the morphology and cellular composition of native intestinal tissue. To model epithelial injury, three common inflammatory stimuli were tested, with TNF-α effectively inducing inflammatory responses consistent with reported organoid phenotypes (58). Further experiments demonstrated that under pathological conditions, CFS reduced inflammatory responses, inhibited epithelial apoptosis, and restored epithelial barrier function. Our observations align with previous studies showing that CFS can mitigate inflammation and enhance barrier integrity across different experimental systems, including both murine models and *in vitro* cell cultures (22). Interestingly, we observed that increasing CFS concentrations did not result in a proportional enhancement of protective effects; rather, high or intermediate doses led to a partial decline in anti-inflammatory and barrier-regulatory efficacy, which may reflect the dose-dependent effects of microbial metabolites or the complex interplay among multiple bioactive compounds present in CFS (59). This is consistent with previous studies using probiotic fermentation supernatants (41). Overall, our findings indicate that within an optimal concentration range, *F. duncaniae* CFS can modulate inflammatory responses and protect epithelial barrier integrity, providing functional evidence linking its depletion to impaired barrier function in diarrheic calves.

Although these results reveal the functional pathways through which *F. duncaniae* may ameliorate colitis, the molecular mechanisms underlying CFS-mediated protection remain incompletely understood. Previous studies have shown that *Faecalibacterium* species exert combinatorial anti-inflammatory effects through multiple parallel mechanisms (60). RNA-seq analysis of organoids co-cultured with 1% CFS revealed modulation of multiple signaling pathways. Notably, genes within the first functional module were primarily associated with NF-κB and chemokine signaling, and the module contained the core regulatory genes, suggesting a key role in immune regulation and inflammatory signaling. Among metabolites produced by *F. duncaniae*, butyrate serves as a critical regulator, modulating epithelial integrity, cytokine production, and T cell differentiation (61) through multiple molecular mechanisms, including HDAC inhibition, NF-κB suppression (62), and GPR/PPARγ activation (63). In our study, 5 mM NaB significantly attenuated inflammatory responses in organoids, promoted epithelial regeneration, and improved barrier function. However, 1% CFS exhibited more pronounced protective effects than butyrate alone, indicating that additional bioactive metabolites in CFS may act synergistically to enhance anti-inflammatory and barrier-restorative responses. The BCM control group showed minimal improvement, further confirming that the protective effects are primarily attributable to microbial secreted products rather than medium components. Beyond SCFAs, *F. prausnitzii* has been reported to produce microbial anti-inflammatory molecules that directly inhibit NF-κB signaling and reduce ROS production in intestinal epithelial cells (64), potentially corresponding to the second functional module identified in our analysis. These molecules also upregulate tight junction proteins such as occludin and ZO-1, reinforcing epithelial barrier integrity and preventing microbial translocation and systemic immune activation. Therefore, the anti-inflammatory effects observed in our study likely result from a multifactorial mechanism involving multiple metabolites, which warrants further investigation.

Our study has several limitations. First, although bovine and ovine gastrointestinal systems are highly similar anatomically and physiologically, potential differences in epithelial immune signaling and barrier regulation may limit direct extrapolation of our results to cattle. Second, the specific active components within CFS and their concentration-dependent effects remain incompletely defined, and the contribution of individual metabolites requires further clarification. Finally, TNF-α represents a single inflammatory stimulus, and other environmental or microbial factors contributing to diarrhea were not modeled. Future studies integrating targeted metabolomics, *in vivo* validation, and single-metabolite intervention experiments are necessary to fully elucidate the protective mechanisms of *F. duncaniae* and its regulatory effects under diverse inflammatory conditions.

In summary, our findings demonstrate that *F. duncaniae*-derived metabolites can preserve epithelial barrier function and mitigate inflammatory damage under pathological conditions through coordinated regulation of multiple signaling pathways. These results provide mechanistic evidence supporting the protective role of *F. duncaniae* in intestinal health and suggest its potential application as a microbial intervention strategy to prevent diarrhea and support epithelial barrier integrity.

### Conclusion

The study successfully developed sheep intestinal organoids capable of long-term passaging, cryopreservation, and resuscitation and established an *in vitro* TNF-α-induced intestinal organoid model. Furthermore, *F. duncaniae*, through its secreted metabolites, modulates key signaling pathways such as NF-κB and chemokine signaling, enhancing epithelial integrity, suppressing pro-inflammatory cytokines, and protecting against epithelial cell apoptosis. These findings not only provide important insights into the potential therapeutic role of *F. duncaniae* in regulating intestinal inflammation and maintaining gut health but also support the translational potential of *F. duncaniae*-derived postbiotics as cost-effective, antibiotic-free nutritional interventions to reduce preweaning diarrhea, improve growth performance, and promote long-term productivity in commercial dairy systems.

## Data Availability

The raw sequencing data of rectal samples from 30 early-stage calves and the raw sequencing data of samples from 40 healthy and diarrheic calves have been deposited in the NCBI Sequence Read Archive under PRJNA1153624. The raw transcriptomic sequencing data have been deposited in the NCBI Sequence Read Archive under PRJNA1401205.

## SUPPLEMENTAL MATERIAL

Fig. S1 aem.00854-26-s0001.tif Construction of an *in vitro* organoid inflammation model.

Fig. S2 aem.00854-26-s0002.tif Through co-incubation, the activation of NF-κB is suppressed and the regulatory and repair pathways are enhanced.

Supplemental legends aem.00854-26-s0003.docx Descriptive legends for Fig. S1 and S2.

Supplemental data sets aem.00854-26-s0004.xlsx Data S1, samples used in this study; Data S2, gene expression and pathway enrichment analysis in response to CON, CFS, and TNF-α treatments.

ASM does not own the copyrights to Supplemental Material that may be linked to, or accessed through, an article. The authors have granted ASM a non-exclusive, world-wide license to publish the Supplemental Material files. Please contact the corresponding author directly for reuse.
