# Supplementary material for: Faecalibacterium duncaniae-derived metabolites protect intestinal epithelial integrity under inflammatory conditions in dairy calves
Source: Appl Environ Microbiol. 2026 Jul 24;92(8):e00854-26. doi: 10.1128/aem.00854-26 (PMC13488333; doi:10.1128/aem.00854-26)
Supplement: Supplementary file 1 [file aem.00854-26-s0003.docx]

**Supplementary Figure 1**. **Construction of an *in vitro* organoid inflammation model.** (A) Organoids (day 6) were treated with different concentrations of DSS for 24 h Scale bars: 100 μm (B) The morphology of organoids after DSS treatment were assessed using optical microscopy (n = 3) (C) Inflammatory cytokine secretion levels after DSS treatment (n = 3) (D) Inflammatory cytokine secretion levels after LPS treatment (n = 3) (E) Organoids (day 6) were treated with different concentrations of LPS for 72 h Scale bars: 100 μm (F) The morphology of organoids after LPS treatment were assessed using optical microscopy (n = 3) Data are presented as mean ± SD One-way ANOVA followed by Dunnett’s multiple comparisons test versus the 0 μg/mL group. ns, no significant difference, * *p* < 0.05, ** *p* < 0.01, *** *p* < 0.001, **** *p* < 0.0001

**Supplementary Figure 2**. **Through co-incubation, the activation of NF-κB is suppressed, and the regulatory and repair pathways are enhanced.** (A) KEGG enrichment analysis based on the pathways associated with the identified genes, following the same size and color significance criteria (Left: CON vs. TNF-α; Right: 1% CFS + TNF-α vs. TNF-α)
